# Circadian disruption, clock genes, and metabolic health

**DOI:** 10.1172/JCI170998

**Published:** 2024-07-15

**Authors:** Lauren A. Schrader, Sean M. Ronnekleiv-Kelly, John B. Hogenesch, Christopher A. Bradfield, Kristen M.C. Malecki

**Affiliations:** 1 Molecular and Environmental Toxicology Center and; 2 Department of Surgery, Division of Surgical Oncology, School of Medicine and Public Health, University of Wisconsin, Madison Wisconsin, USA.; 3 Divisions of Human Genetics and Immunobiology, Department of Pediatrics, Cincinnati Children’s Hospital Medical Center, Cincinnati, Ohio, USA.; 4 Department of Oncology and; 5 Department of Population Health Sciences, School of Medicine and Public Health, University of Wisconsin, Madison, Wisconsin, USA.; 6 Division of Environmental and Occupational Health Sciences, University of Illinois Chicago, Chicago, Illinois, USA.

## Abstract

A growing body of research has identified circadian-rhythm disruption as a risk factor for metabolic health. However, the underlying biological basis remains complex, and complete molecular mechanisms are unknown. There is emerging evidence from animal and human research to suggest that the expression of core circadian genes, such as circadian locomotor output cycles kaput gene (*CLOCK*), brain and muscle ARNT-Like 1 gene (*BMAL1*), period (*PER*), and cyptochrome (*CRY*), and the consequent expression of hundreds of circadian output genes are integral to the regulation of cellular metabolism. These circadian mechanisms represent potential pathophysiological pathways linking circadian disruption to adverse metabolic health outcomes, including obesity, metabolic syndrome, and type 2 diabetes. Here, we aim to summarize select evidence from in vivo animal models and compare these results with epidemiologic research findings to advance understanding of existing foundational evidence and potential mechanistic links between circadian disruption and altered clock gene expression contributions to metabolic health–related pathologies. Findings have important implications for the treatment, prevention, and control of metabolic pathologies underlying leading causes of death and disability, including diabetes, cardiovascular disease, and cancer.

## Introduction

The daily routines of life on earth are bound to the patterns of the natural environment, leading to the evolution of circadian rhythms in nearly all organisms (1). In animals, circadian rhythms are the daily, timed cycles that coordinate biological functions and prepare the body for recurring diurnal activities such as sleeping, eating, and physical activity (2). Cues such as sunlight, food, sound, and temperature, called zeitgebers, attune circadian rhythms to external conditions, coordinating basic cellular functions to match the environment and maintain homeostasis. A large body of evidence now links an increasing number of known circadian disruptors or zeitgebers, including sleep/wake patterns, dietary timing, and caloric intake, to adverse outcomes in humans. A comprehensive look at evidence from both human and animal studies exploring the relationship between circadian gene expression and metabolic health is lacking. Consequently, we lack a clear mechanistic understanding necessary for future prevention, treatment, and control of metabolic health–related diseases, including diabetes, cancer, and cardiovascular diseases, the leading causes of death and disability across the globe.

Here, we aim to summarize evidence from in vivo animal models and compare these results with epidemiologic research findings to advance understanding of mechanistic links between circadian disruption and altered circadian locomotor output cycles kaput gene (clock) gene expression contributions to metabolic health–related pathologies.

**Figure 1:**
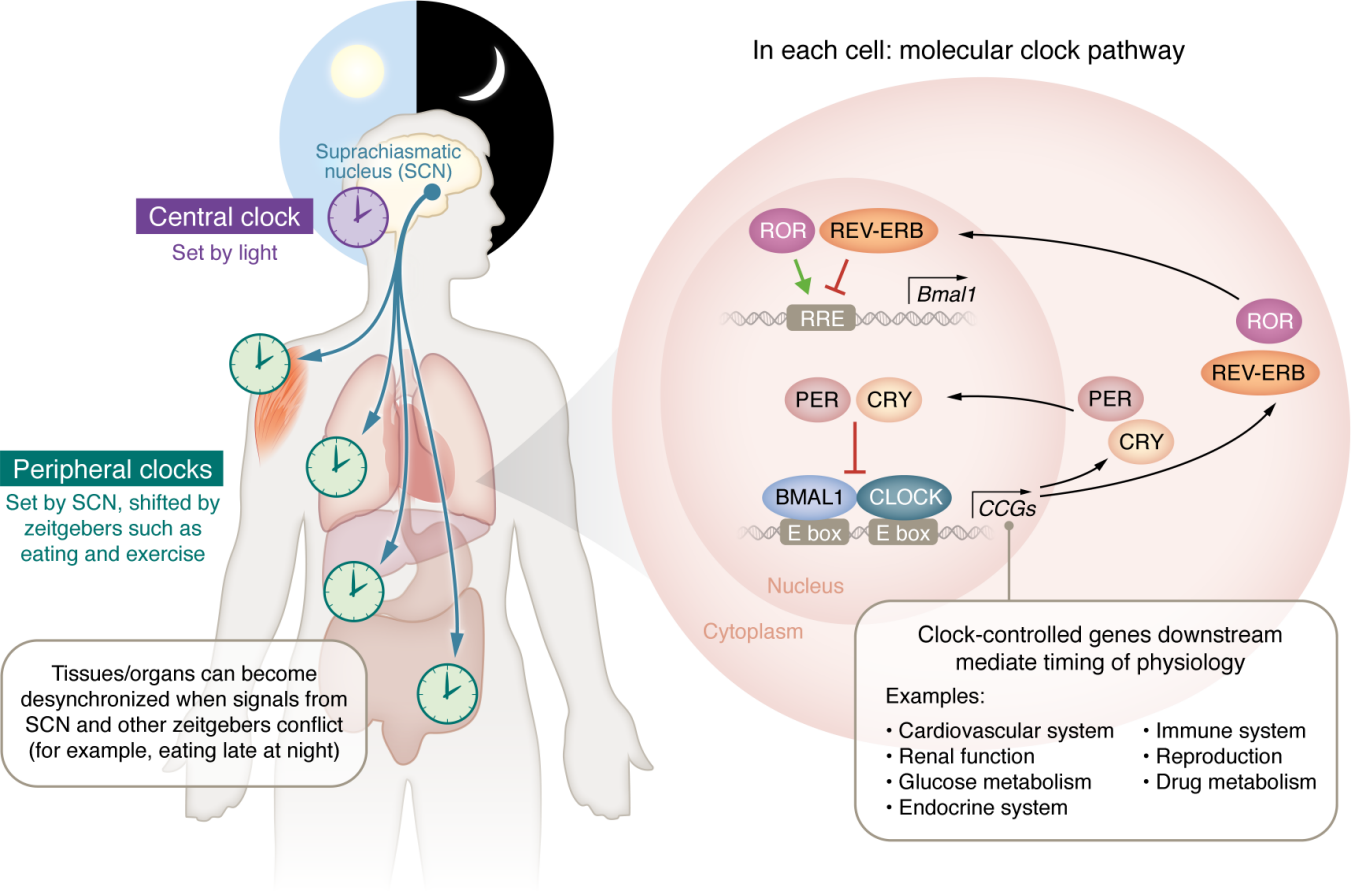
Circadian control of molecular core clock gene signaling and physiologic regulation. The central, peripheral, and molecular clocks and the physiological processes under circadian control. The circadian clock (purple) in the suprachiasmatic nucleus (SCN) of the brain sets peripheral clocks in individual organs and tissue types (light green) via signals including circulating hormones, metabolites, the sympathetic nervous system, and body temperature. Within the cells of the SCN and each organ/tissue type, each cell contains transcription-translation feedback loops, the molecular clocks that drive circadian rhythms. These molecular clocks regulate the transcription of thousands of CCGs and direct the daily oscillatory expression of thousands of COGs and additional transcription factors that mediate the timing of myriad physiological processes as represented in the molecular clock pathway within cells.

At a cellular level, core clock genes (CCGs) direct the daily oscillatory expression of thousands of clock output genes (COGs) (Figure 1). This complex system regulates a myriad of physiological processes, synchronizing them to match diurnal needs (3, 4). Many of these output genes are associated with other nuclear receptors found throughout metabolic tissues (5). The molecular clock is driven by a dimer of the CLOCK and brain and muscle ARNT-Like 1 gene (*BMAL1*) (also known as MOP3 and ARNTL, respectively) transcription factors (6, 7), which binds to genomic response elements known as E-boxes, regulating the expression of thousands of genes, including the period (*PER*) genes (*PER1, PER2*, and/or *PER3*) and cyptochrome (*CRY*) genes (*CRY1* and *CRY2*) (8). In turn, the PER and CRY proteins accumulate in the cytoplasm and, later in the cycle, translocate to the nucleus, where they suppress the CLOCK:BMAL1 dimer, reducing their own expression (4). Still later in the cycle, PER and CRY are degraded, CLOCK and BMAL1 expression resumes, and the process repeats with a 24-hour rhythm (9, 10). The CLOCK:BMAL1 dimer also influences the rhythmicity of the molecular clock through the E-box–dependent regulation of additional transcription factors, such as RORA, RORB, RORC, NR1D1 (REVERBA), and NR1D2 (REVERBB). The ROR transcription factors stimulate BMAL1 transcription, and REVERB transcription factors suppress it, creating a second loop that reinforces the E-BOX loop. Together, this transcriptional-translational feedback loop (TTFL) sets off a cascade of transcriptional events that can influence the rhythmic expression of up to 25% of the human genome (2, 4, 9, 11).

In modern society, humans have become less constrained by nature’s day/night cycles, and circadian rhythms are pushed and pulled by inconsistent zeitgebers. These circadian disruptors include artificial light, continual food availability, ever-changing work and social demands on sleep/wake timing, and, more recently, diet and timing of food intake. This disruption is especially pronounced for the approximately 15% of the US workforce who work nondayshift schedules, at least 35% of whom live in constant sleep deprivation (12, 13). Moreover, studies also report that almost 70% of working adults operate in states of “social jet lag” (14), where circadian disruption occurs as a result of an individual’s shifting or shortening of sleep on weekdays, followed by additional sleep on weekends (15).

When organisms are exposed to circadian disruptors, many biological systems and feedback loops meant to anticipate and regulate diurnal homeostasis can become misaligned through previously described feedback loops at the molecular and cellular levels. Over time, this dysregulation may negatively affect organs and tissues such that catabolic and anabolic processes are out of sync, leading to pathological consequences. For example, aberrant timing of zeitgebers, such as eating during the inactive phase, may induce (a) a generalized environmental desynchrony where the systemic clock is out of phase with external cues such as food timing or (b) an “internal” circadian desynchrony of peripheral clocks (e.g., liver, pancreas) and the central clock, both scenarios with the potential to reduce the efficiency of energy metabolism, leading to weight gain (e.g., see refs. 16–23) and downstream effects on other metabolic pathways. Thus, through regulatory feedback mechanisms, exposure to circadian disruptors may contribute to the development of human disease or inflict adverse health outcomes via inappropriate phase relationships between the internal tissue clock and environmental cues (24–27). Despite relatively rapid rates of behavioral reentrainment (e.g., sleep patterns) following disruption, internal organs reentrain at different rates, adding additional complexity to the potential impact of circadian desynchrony on human health and disease (28–31). This latter point may be particularly relevant for humans with inconsistent periods of active/inactive cycles over the long term (e.g., shift workers alternating between day and night shifts). Such individuals may rarely reach a state where systemic or tissue clocks are in sync with their external environment, especially energy consumption.

**Figure 2:**
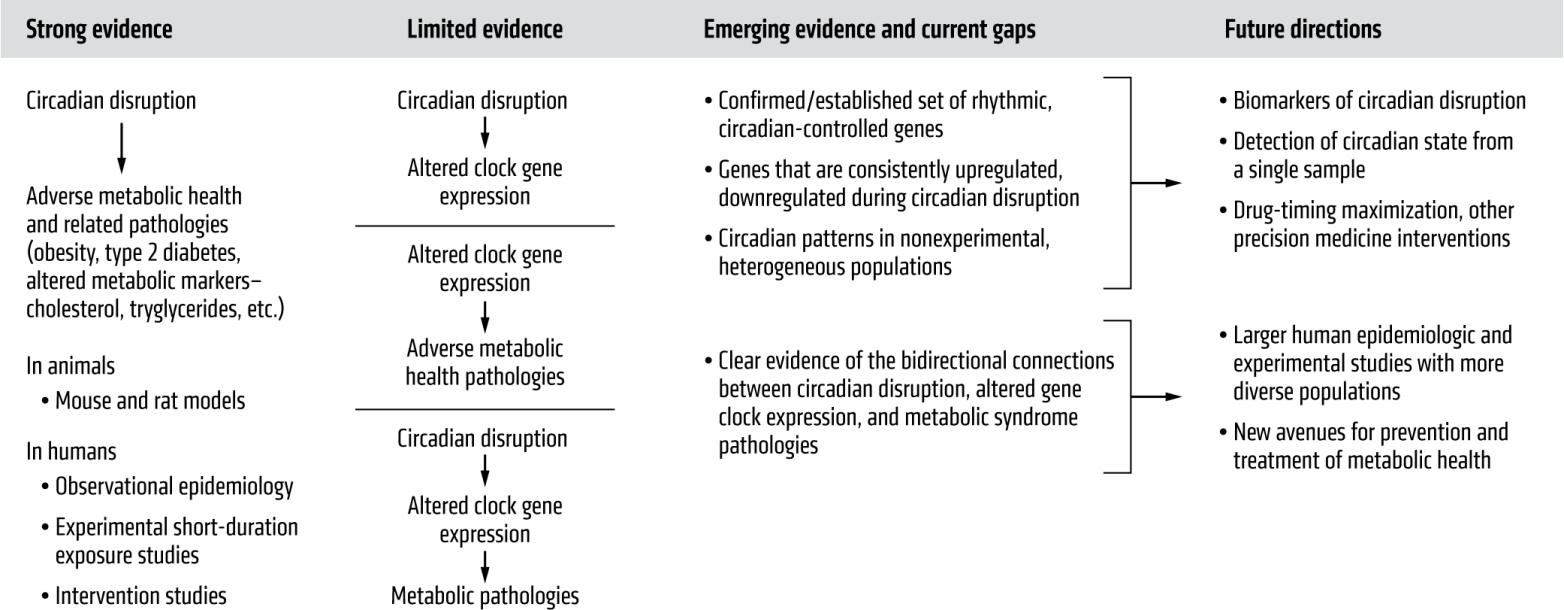
Foundational evidence of core clock gene regulation and metabolic health in animal models and human studies. Summary of the current state of knowledge regarding circadian disruption, CCG expression, and the development of adverse metabolic health–related pathologies.

Circadian disruption has been studied as a contributor to the development of the constellation of metabolic health–related pathologies, including obesity, diabetes, and metabolic syndrome (MetS), a cluster of indicators including abdominal obesity, high blood pressure, high blood sugar, high serum triglycerides, and low serum high-density lipoprotein (HDL) cholesterol (32). Early support for this relationship came from both animal models of circadian disruption and human studies of shift workers (33–37). This research suggests that exposure to circadian disruptors leads to mistimed or dampened expression of CCGs and COGs, altering metabolic regulation and contributing to adverse metabolic health and related pathologies (26). Here, we update the current understanding of these phenomena by examining the evidence linking circadian disruption with clock gene expression and metabolism in animal and human studies to provide a foundation for future translational and clinical research applications as summarized in Figure 2 (38, 39).

## Circadian disruption, clock genes, and metabolism

### In vivo animal models of circadian rhythms.

Early investigations into circadian rhythms relied on easily measured physiological aspects of circadian rhythms, such as body temperature and locomotor activity (40, 41). In animals, genetic drivers of circadian rhythms were first revealed through forward genetic screening in *Drosophila melanogaster,* which identified mutants with short, long, and arrhythmic circadian locomotor activity patterns (40, 41). These studies revealed that the *per* gene and its protein product PER, a founding member of the PER-ARNT-SIM (PAS) superfamily, was an essential component of the circadian clock. Subsequent studies in mammals confirmed these *PER* findings and led to the discovery of additional CCGs, *CLOCK*, *BMAL1*, and *CRY1/CRY2,* as well as three orthologs of *Drosophila*: *PER (PER1/PER2/PER3)* (37, 38). Notably, CLOCK, BMAL1, and PER were all identified as members of the PAS family of proteins, suggesting hetero- and homodimeric interactions driven by similar domain structures lie at the mechanistic heart of the molecular clock (Figure 1) (5, 36, 37, 42–45).

As mutant models were generated in mice, the circadian clock’s role in metabolic health–related pathologies emerged. Table 1 shows an overview of select studies providing evidence that clock genes are linked to metabolic health pathologies in animal models. For example, in an experiment with mice mutant for the *Clock* gene, mutants displayed altered food intake timing with ad libitum feeding, consuming more calories outside the active phase (37). These animals also had dampened activity rhythms and developed obesity and MetS, including high cholesterol, high triglycerides, high blood sugar, hypoinsulinemia, and elevated leptin during the rest phase. Similar metabolic perturbations were seen in other mouse models with induced mutations in molecular clock components. For example, *Bmal1*-null mice displayed blunted postprandial insulin responses, decreased gluconeogenesis, and loss of typical glucose and triglyceride rhythms (36, 45). Likewise*, Per2*-mutant mice developed without normal glucocorticoid rhythms (46) and *Cry1/Cry2* double mutants displayed altered liver metabolism and altered patterns of circulating growth hormone (44). Finally, when compared with WT, transgenic mice generated to overexpress *Cry1* exhibited treater hyperglycemia without increased weight gain (47).

Further examinations designed for understanding the relationship between clock and glucose homeostasis in *Clock-*mutant mice have revealed altered expression of genes involved in pancreatic islet cell development and insulin signaling (45). At eight months, these mutants displayed elevated serum glucose compared with WT counterparts, seemingly consequent to defective glucose-stimulated insulin release from the *Clock*-mutant islets. Interestingly, findings from young *Clock*-mutant mice aged two to three months demonstrate that a compensatory component exists early on, driven by the *Clock* mutation in other organ systems such as the liver, leading to age-related emergence of insulin resistance and underlying deficits in insulin secretion manifested as pathogenic hyperglycemia (i.e., diabetes) (45). An explanation for the age-dependent hyperglycemia phenotype may come from the observation that *Clock*-mutant and *Bmal1-*null mice exhibit loss of typical glucose and triglyceride rhythms, with impaired and abolished gluconeogenesis, respectively (36). Because gluconeogenesis occurs predominantly in the liver, the contrasting effects between the paired metabolic organs regulating glucose homeostasis in *Clock*-mutant and *Bmal1-*null mice caused a “masking” of the phenotype when the animals were young. Examples such as this highlight the challenges in disentangling the role of the circadian clock when using global mutant/null models (36).

To address this issue, pancreas-specific *Bmal1*-null mice (*Bmal1^fl/fl^; Pdx1-Cre*) were employed to knock out the circadian clock in pancreatic islet cells. These experiments revealed that, even when mice are at a young age, the islet cell clock influences insulin secretion, glucose levels, and glucose tolerance (i.e., hypoinsulinemic diabetes) (45). Thus, the discrepancy in young versus old *Clock*-mutant mice may be due to the circadian clock yielding different tissue-specific effects in metabolic processes such as glucose metabolism, which is particularly relevant for the pathologic processes of diabetes and MetS. Further, many metabolic conditions emerge in humans with aging, e.g., type 2 diabetes (T2D), nonalcoholic steatohepatitis, etc. Concordantly, disruption of the circadian clock in humans results from global disruption/desynchrony. Therefore, it is plausible that the early impact of circadian desynchrony on MetS in humans is difficult to completely understand given the organ-specific functions of the clock. In fact, disease states may only become apparent after long-standing desynchrony, which is supported by studies demonstrating increased risk of developing diseases such as diabetes with increasing duration of shift work (48–51).

Glucose homeostasis is influenced by cellular-specific gene signaling mechanisms controlled by specific CCGs. For example, a 2015 study by Perelis et al. examined pancreatic β cells from mice with intact or disrupted BMAL1 expression. For intact cells, CLOCK/BMAL1 dimers were shown to bind to regulatory sites (CCGs) in islet cells to drive transcription of genomic targets (COGs) in these cells that were distinct from those of other cells in the liver. They further found that mice with disrupted BMAL1 expression developed glucose intolerance, suggesting a direct genetic mechanism controlling diabetes as one measure of metabolic health (52). Further, the majority of CLOCK/BMAL1 binding sites identified within β cells are not commonly identified in the liver or other tissues — supporting the tissue-specific role of the clock in altering metabolic health through active enhancer regions and epigenetic chromatin regulation of unique genes within cells and suggesting that polymorphisms or alterations contribute to metabolic disruption. Tissue-specific subsets of circadian genes reinforce this idea. Despite the substantial number of cycling genes in each tissue (5), only a small number of common genes are rhythmic in all tissues (11).

A large body of work has been undertaken for understanding the consequences of external/environmental circadian disruptors in mice (53). These protocols are meant to mimic circadian disruptors commonly experienced by humans, such as altered timing of light exposure, activity, sleep, or food intake (17, 30, 54–56). Experimental studies aiming to mimic shift-work exposures through phase-shift and time-restricted feeding protocols suggest that exposure to these various circadian disruptors can alter metabolic health and CCG expression (56–58). A 2011 study found that phase-shift protocols among Sprague-Dawley rats were associated with changes in the acceleration of multiple indicators of T2D, particularly for animals with altered β cells (58). Similarly, a 2021 study of circadian disruption via chronic jet lag investigated transcriptional changes in mice and found that 5% of the transcriptome in the pancreas is regulated by CCGs and that external phase shifting in mice alters regular rhythmic control of genes in the pancreas associated with insulin and enzyme regulation (56).

Emerging evidence from experimental food-intake and timing models illustrates the multiple mechanisms by which circadian disruption affects metabolic health. For example, WT mice subjected to misaligned food intake relative to active and inactive periods display accelerated weight gain similar to that of humans. Along with lowered amplitude of clock genes, mice consuming a high-fat diet during their inactive period gained substantially greater weight and had a higher body fat percentage than mice consuming the same diet during their active period (18). In contrast, when a time-restricted feeding (TRF) protocol restricted feeding to the active period, mice on high-fat diets were protected from weight gain and the increased markers of adverse metabolic health experienced with ad libitum feeding. In several studies, TRF prevented obesity and impaired glucose tolerance, restored insulin sensitivity, and protected against inflammation and hepatic steatosis (59–61). Moreover, during the active phase, TRF restored normal hepatic glucose metabolism elicited by the ad libitum high-fat diet. TRF also restored the oscillation of metabolic regulators in the liver that were dysregulated with an ad libitum high-fat diet. Moreover, it restored CCG expression amplitude. Thus, eating at times misaligned to circadian rhythms leads to altered metabolic health, while TRF ameliorates this pathology.

A combination of TRF and diet quality affects metabolic health by regulating CCGs, as seen in a series of critical mouse experiments. Animals were subjected to high-fat diets with either ad libitum feeding, TRF confined to an active period, or TRF confined to an inactive period (59). Despite similar activity and timing of food intake overall, a high-fat diet during the inactive period caused increased body mass and lower energy expenditure. Carbon-labeling studies demonstrated that the high-fat diet during the inactive period resulted in decreased glycolysis in adipocytes and dampened oscillation of CCGs compared with the high-fat diet during the active period. A series of genetically engineered mouse models demonstrated that the adverse metabolic consequences of a high-fat diet during the inactive period were related to impaired adipocyte thermogenesis. The mediators of thermogenesis in the adipocytes were found to be regulated by the core molecular clock and responsible for maintaining metabolic health in the high-fat diet/active-phase TRF group. This study supports the idea that optimal metabolic health can depend on the alignment of food intake and the biological rhythms of cellular thermogenesis controlled by the molecular clock. Moreover, these data help us to understand the contributions of high-risk dietary regimens and circadian misalignment. These results also underscore important considerations for humans experiencing circadian disruption, leading to the suggestion that mitigation of risk for adverse metabolic health could include strategies to align specific nutrient intake with the internal clock and highlighting the importance of developing simple and reproducible methods of monitoring organ-specific reentrainment that may facilitate optimizing such an approach.

### Foundational epidemiologic and population-based research findings.

While human studies often lack the specificity to advance mechanistic insights, coupled with animal-based findings, they provide crucial foundational evidence linking circadian disruption to metabolic health, as summarized in Table 2. Some of the first studies to suggest circadian disruption alters metabolic health come from epidemiological investigations of shift workers. For example, an examination of 54,724 participants in the Nurses’ Health Study II found that individuals exposed to any duration of night-shift work had increased odds of obesity, higher total calorie intake, and shorter sleep durations than those who had never worked night shifts, after adjusting for age and socioeconomic status (62). In a separate study, shift workers displayed increased odds of being overweight or obese and were more likely to report insufficient sleep than individuals working traditional schedules. Moreover, there was a stronger association between shift work and overweight conditions among individuals reporting insufficient sleep, suggesting a protective effect of adequate sleep duration during shift work (63). A much smaller study of 24 women (12 night-shift and 12 day-shift workers) found that night-shift workers had greater fat mass, larger energy intake, impaired sleep, lower insulin sensitivity, and higher triglycerides compared with their day-worker counterparts (64). Night-shift workers also had higher postprandial ghrelin levels and lower bloodstream levels of xenin, a gut-derived hormone, offering preliminary evidence of shift work and metabolic health in the form of appetite regulation (64).

Another significant circadian disruptor linked to metabolic health is “social jet lag,” measured as the difference in midsleep time on nights before work or school and those before work- or school-free days (14). One study of 815 nonshift workers born between 1972 and 1973 in New Zealand identified an association between social jet lag and numerous metabolic health indicators, including BMI, fat mass, and waist circumference (65). In independent studies, individuals with social jet lag displayed greater adiposity, lower HDL cholesterol levels, higher triglycerides, increased insulin resistance, and higher fasting plasma insulin even after controlling for behavior and sleep quality. Moreover, individuals with a tendency to be most active in the evenings and delay sleep onset, known as “evening chronotypes,” had lower HDL cholesterol levels, consistent with similar findings that evening types have a heightened risk for cardiovascular disease, given their proclivity to circadian-disrupted schedules and social jet lag in particular (66, 67).

Additional insights come from several studies among non–shift-work female populations. A cross-sectional analysis of non–shift-working middle-aged women revealed a positive association between bedtime variability and bedtime delay with increased insulin resistance (68). After more than five years of follow-up, greater bedtime delay predicted higher insulin resistance, suggesting that both acute- and chronic-inconsistent sleep timing induce metabolic dysfunction (68). These observations were echoed among a study of older women over the age of 80 that found intraindividual variation in objectively measured wake time, sleep duration, sleep delays (social jet lag), and the midpoint of sleep were associated with alterations in body composition, including percentages of increased fat mass and lower lean mass as metabolic health indicators (69).

Experimental phase-shift studies among adults requiring short-term alterations of regular sleep patterns provide additional evidence for links between circadian disruptors and markers of metabolic health. In one such study, 21 healthy adults (10 men and 11 women) of varying ages were subjected to circadian disruption, achieved via imposed 28-hour days and 5.6 hours per night sleep restriction. After three weeks, circadian-disrupted participants had increased fasting and postprandial glucose levels and significantly decreased resting metabolic rates compared with baseline, a pattern observed in both young and old subjects. These changes normalized after nine days of return to standard sleep/wake patterns (70). In a separate study, two experimental groups of healthy young adults were exposed to two five-day weeks of five hours per night of sleep restriction, with one group permitted an interim two-day weekend of ad libitum “catch-up” sleep, while the other group continued sleep restriction for the entire study period. A control group with normal sleep (nine-hour daily sleep windows) was also included. Despite sleeping an extra three hours each weekend day, the weekend catch-up group experienced reduced insulin sensitivity relative to controls. While all three groups increased their energy intake compared with baseline calorie-controlled meals, only the control group did not experience weight gain. Moreover, both disrupted groups increased after-dinner snacks on days following sleep restriction. These findings suggest that a weekend catch-up sleep after sleep debt, akin to social jet lag, is not sufficient to correct the metabolic dysfunction accrued during sleep restriction (71).

There is mounting evidence that TRF, particularly TRF during the active phase and aligned with circadian rhythms, is also beneficial for human metabolic health, which is similar to observations in mice. A meta-analysis of 19 TRF clinical trials found TRF diets significantly reduced body weight, decreased fat mass, preserved fat-free mass, and reduced systolic blood pressure, triglycerides, and fasting glucose (72). TRF is thus seen as a promising treatment for changing metabolic health, even without reducing the total calorie intake, hence emphasizing the importance of circadian timing in metabolic processes (72). Further, in a disrupted sleep and TRF study, participants with three days of 28-hour day simulated night-shift protocols were divided into two groups and fed meals in sync with their shifted/disrupted schedule or their usual dietary intake schedule. The group fed in sync with their shifted/disrupted, 28-hour cycle (eating meals between midnight and 4 am, when they would typically be asleep) showed impaired glucose tolerance (73). In parallel with experimental animal findings, human study results further establish the role of food intake as a strong entraining cue for the circadian clocks in the periphery and indicate metabolic outcomes can be improved by aligning the food-intake window with the active phase while removing windows of food intake later in the day or night.

### Circadian disruption and CCG expression.

Circadian disruption is also associated with altered clock gene expression in human studies, offering clues to the biological mechanisms linking circadian disruption with metabolic health. A small 2019 study including 18 female nurses revealed fewer rhythmic genes observed in the PBMCs of rotating night shift workers (*n* = 9) compared with nurses working day shift (*n* = 9). Moreover, phase desynchrony of core body temperature, peak cortisol, and dim-light melatonin onset were also observed (74).A more extensive investigation of 60 nurses using a single measurement time point identified differential expression of nearly all CCGs, including transcripts of the CCGs *BMAL1, CLOCK, NPAS2, PER1, PER2, PER3, REVERBA, CRY1*, and *CRY2* genes. These CCGs were dysregulated in morning blood draws taken among rotating shift nurses and compared with those day-shift nurses (75). A small study of eight participants exposed to a simulated night-shift work protocol, including three days of ten-hour sleep period delay, repeated blood draws over two 24-hour periods, and transcriptome-wide analysis of PBMCs, showed a significant reduction of rhythmic transcripts, including *PER3* postdisruption compared with baseline (76).

Interestingly, similarly to what was found in the 2019 nurses’ study (74), some CCGs, including *PER1* and *BMAL1,* maintained rhythmicity. Genes that remained rhythmic after disruption displayed dampened oscillation patterns that matched habitual sleep and wake times rather than the newly disrupted schedule. These results show that, while circadian function may remain largely intact in a simulated night shift, many COGs lost expression amplitude, influencing several important metabolic pathways, including immune-system regulation (76).

In a separate forced desynchrony experiment, 22 participants underwent baseline assessment followed by several 28-hour days of forced desynchrony, with sleep onset pushed back four hours further each night. During disruption, 24-hour melatonin rhythms remained largely preserved as compared with baseline rhythms. In contrast, there was a six-fold reduction in PBMC-measured circadian transcripts, including *BMAL1, CLOCK*, and *PER3* (77). Experimental sleep-deprivation studies also aimed at identifying resultant transcriptome alterations have identified similar reductions in the circadian rhythmicity of genes and changes in the expression of genes from chromatin-remodeling immune and stress-response pathways. In a study of 26 sleep-restricted individuals who were limited to six hours per night, gene expression in thousands of transcripts was altered, and the number of genes with detectable circadian patterns was reduced by 20%. Again, *CLOCK, PER1, PER2, PER3, CRY2*, and *RORA* were significantly impacted. Additionally, several metabolic and oxidative stress–related genes were altered after the sleep-restriction protocol (78). It is important to note that these transcriptome-wide analyses were conducted on relatively small numbers of individuals with short desynchrony and sleep-deprivation protocols. Therefore, the longer-term impacts of these exposures are unclear. Nonetheless, these findings collectively support the idea that circadian misalignment has widespread effects on the transcriptome and has a differential impact on central versus peripheral clocks, highlighting the potential for adverse metabolic health from disruption of the habitual sleep/wake cycle (see a summary of findings in Table 3).

### CCGs and metabolic pathology.

The connection between clock gene expression and metabolic health may be bidirectional. Evidence from various human studies suggests that a state of adverse metabolic health itself, including elevated BMI, can alter the expression of CCGs in a tissue-specific fashion. In one study of 21 lean and 28 morbidly obese female nonshift workers without diabetes, examination of 24-hour adipose gene expression revealed that obese individuals displayed altered circadian expression of many CCGs, including *CLOCK, BMAL1, PER1, CRY2,* and *REVERBA*, compared with healthy, lean subjects. Positive correlations were found among all subjects between *REVERBA* and BMI/waist circumference, CLOCK and LDL cholesterol, and *RORA* with HDL cholesterol. An interesting conclusion from this study was that *REVERBA* is an important gene associated with metabolic health (79).

Further evidence of connections between metabolic phenotypes driving circadian disruption comes from studies showing that weight loss alters CCG expression patterns. A 2020 study examined differential mRNA levels and expression of CCGs in skeletal muscle among 23 obese patients (5 women and 18 men) undergoing gastric bypass surgery and 14 normal-weight controls (6 women and 8 men). Males in the obese group had significantly lower *CLOCK, CRY1*, and *CRY2* expression than lean male controls (80). Obese women exhibited downregulated *CRY1* mRNA levels compared with lean female controls, but *CRY1* expression was restored to lean-control levels following gastric bypass–induced weight loss (74). Interestingly, while changes in CCG expression varied by sex, additional research is needed to replicate these findings. A second study examined participants’ expression of CCGs in adipose tissue before and after hypocaloric diet–induced weight loss (81). After eight weeks, 50 subjects who lost 8% or more of their body weight saw significant increases in *PER2* expression compared with baseline, with similar changes to genes regulating fat metabolism, autophagy, and inflammatory responses (81).

Human studies also suggest that even limited, short-term exposure to circadian disruptors can alter metabolic pathways and clock gene expression. One such study, in 2018, subjected 14 healthy men to three days of normal sleep, followed by three days of reversed day/night schedules. After three days of disruption, fasting glucose and free fatty acids were significantly elevated compared with what occurred with normal sleep conditions (82). In addition, a significant transcriptional alteration in PPAR signaling was observed, leading to the hypothesis that misalignment promotes a preference for intramuscular fatty acid metabolism over glucose metabolism. Interestingly, after the three days of misalignment, CCGs had not reentrained to the reversed schedule and remained aligned to the regular day/night schedule (82). In another 2015 investigation, 15 healthy male participants were exposed to acute 24-hour sleep deprivation, and increased methylation in the *CRY1* and *PER1* genes in adipose tissue was observed, suggesting that methylation is also a mechanism for the downregulation of CCG expression (83). Furthermore, after sleep deprivation, expression of *BMAL1* and *CRY1* in skeletal muscle was decreased, and postprandial plasma glucose concentrations were increased (83). These studies also provide evidence that even short-term misalignment of the circadian clock from standard behavior patterns, from a single night of wakefulness to a few days of misalignment, can be linked to metabolic changes in humans.

Studies of CCG expression have also shown that TRF studies can help combat circadian misalignment’s negative consequences, suggesting new opportunities for preventing and treating adverse metabolic health outcomes. In a 2019 crossover study of 11 obese participants, the efficacy of TRF was investigated (84). Comparison of four-day ad libitum feeding (8 am–8 pm) with an early daytime feeding window (8 am–2 pm) revealed that TRF resulted not only in increased expression of CCGs (*BMAL1, CRY1, CRY2*, and *RORA*), but also elevated ketones, elevated cholesterol levels, reduced mean blood glucose levels, and reduced glucose spikes throughout the day. This was despite equal calories consumed between conditions. While many studies have supported using TRF to improve metabolic health, this experiment provided key insights by measuring and associating CCG expression with improvements in lipid metabolism and glucose regulation (84). A similar randomized case-crossover study of TRF examined 11 men who were either overweight or obese and found that TRF improved daytime insulin profiles and reduced night-time glucose levels. The oscillation patterns of CCGs, including *CLOCK, BMAL1, CRY1*, *PER1*, -*2*, and -*3*, and *REVERBA* and -*B,* were unchanged between 15-hour free-feeding and 8-hour TRF conditions (85). However, the authors identified an increase in the amplitude of oscillating muscle transcripts related to amino acid transport, suggesting that TRF has multiple health benefits (85).

As in mice, genetic variation in CCGs and genes that modify the clock can influence rhythms and metabolic health in humans. For example, in a meta-analysis of cohort studies from the Cohorts for Heart and Aging Research in Genomic Epidemiology Consortium, associations among sleep duration, genetic variants of core clock and other circadian genes, and cardiometabolic traits were identified (86). In carriers of the T allele of the melatonin receptor 1B, long sleep duration (9+ hours per night) was associated with increased BMI. Additionally, in carriers of the A allele of *CRY2* (SNP rs11605924), sleep duration was positively associated with HDL cholesterol level (86). Additional evidence supporting the connection can be seen in investigations of SNP mutations and their role in metabolic health, reviewed in Škrlec et al. (87). The growing evidence of CCG SNPs underlines the complex relationships between CCGs and metabolic health. Table 4 summarizes several examples and study findings.

## Conclusions

Research across organisms supports th idea that CCGs and clock-controlled genes control link circadian disruption and metabolic health, including MetS and diabetes. Specifically, there is a substantial and growing body of evidence from CCG mutant mouse models linking clock activities to pathological metabolic outcomes. Tissue-specific CCG mutant mouse models have also helped elucidate the effects of CCGs in each organ and tissue type. Moreover, TRF protocols in mice offer strong evidence for food intake as a zeitgeber/disruptor and consequential CCG changes that have metabolic pathological consequences.

From a risk paradigm, circadian disrupter impacts are a consequence of their quality and exposure duration. Alternating shift work (as defined by discrete and alternating periods of daytime, swing, and night work) provides an example of this concept. In a tissue desynchrony model, the oscillating metabolic profiles of distinct tissues are out of proper alignment due to the differential response times required to reestablish normal rhythms after an inappropriate zeitgeber exposure or significant circadian perturbation. Extended periods of alternating shifts may not allow for appropriate internal organ reentrainment. This has potential relevance in alternating shift workers and other highly dyssynchronous populations, such as frequent international travelers or with weekly social jet lag. The continual shift in zeitgebers likely requires a unique strategy to optimize realignment and promote a positive impact on health, including mitigation of adverse metabolic pathologies.

It may also be important to emphasize that shift work is not always synonymous with circadian desynchrony. One can predict the existence of many shift workers with appropriate circadian hygiene that allows alignment of the timing of nutrient intake with internal physiologic processes (88). The challenge lies in how to disentangle those components of circadian misalignment contributing to poor human health, given the potential for synergy, additivity, or antagonism for a spectrum of prevalent disruptors such as extended timing of food intake (i.e., eating outside the active phase) and extended exposure to light at night (screen time) intertwined with poor sleep habits.

With these ideas in hand, it becomes possible to more precisely define circadian disruptors as those risk factors that lead to circadian desynchrony. In a simple example of this idea, circadian disruption results from the inappropriate timing of a zeitgeber, which can alter the clock phase, decrease its amplitude, and disrupt synchrony in core clock factors and outputs across tissues. An important consequence of this definition is that many apparent risks can be misidentified or misdiagnosed. Take the “shift worker” designation. In one extreme, a subpopulation of shift workers may pay remarkable attention to circadian hygiene and may shift zeitgebers such as artificial light and mealtimes in accordance with their shifted schedule. In such a case, this subpopulation may not experience any circadian disruption despite being shift workers. The focus then becomes not just specifically modulation of sleep patterns or shift-working paradigms but, more importantly, those critical behavioral patterns, such as the timing of nutrient intake in alignment with the appropriate phases and amplitude of the internal clock (projected as the active phase).

Another critical consideration is the organ-specific function of the circadian clock and the distinct time to reentrainment of the clock behaviorally and in various organ systems following a circadian disruption. Internal organ reentrainment may not be mirrored by behavioral realignment, given the discordance identified between time to reentrainment in circadian behavioral patterns (activity/rest) and metabolic organ circadian rhythms (e.g., pancreas). This lends support to the value of cataloging predictably oscillating CCGs — or downstream clock-controlled genes — as useful biomarkers to determine an individual’s internal circadian time. Such biomarkers would be invaluable in circadian-rhythm research, circadian-targeted drug dosing, clinical diagnostics, and epidemiology to identify at-risk populations. Various groups have undertaken this challenge, utilizing artificial intelligence and machine learning applied to a range of biological sample types in both mice and humans, including whole blood (89–91), monocytes (92), hair-follicle cells (93), skin (94), breath (95), and multiorgan tissue sampling from autopsy (96).

In parallel, human studies offer mounting evidence linking circadian disruption to metabolic health, with a wide range of study types echoing and complementing each other’s findings in analogous animal studies. However, existing studies are limited by the inherent nature of human subject research, lack of diversity in sample size, and lack of long-term studies in real-world settings. Nonetheless, phenotypic associations between numerous metabolic health outcomes and circadian disruptors, including circadian misalignment backed up by mechanistic animal studies, support the notion that CCGs are important systemic regulators in metabolic disorders. Future human research is needed to expand this evidence base and further illuminate the details of these relationships.

## Figures and Tables

**Table 4:**
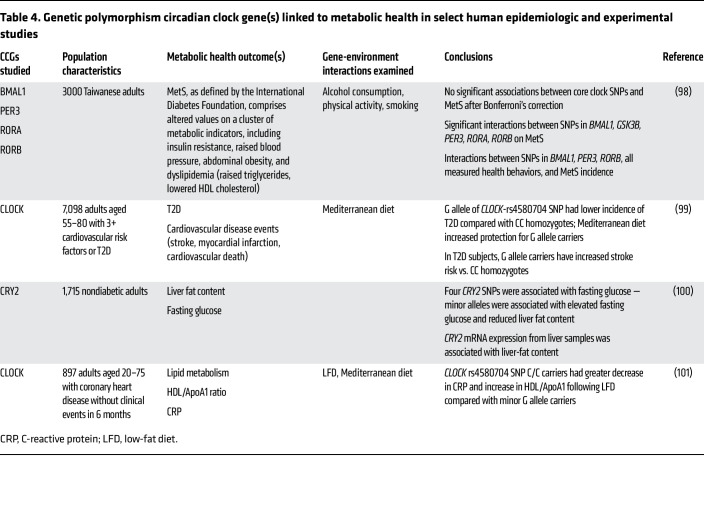
Genetic polymorphism circadian clock gene(s) linked to metabolic health in select human epidemiologic and experimental studies

**Table 3:**
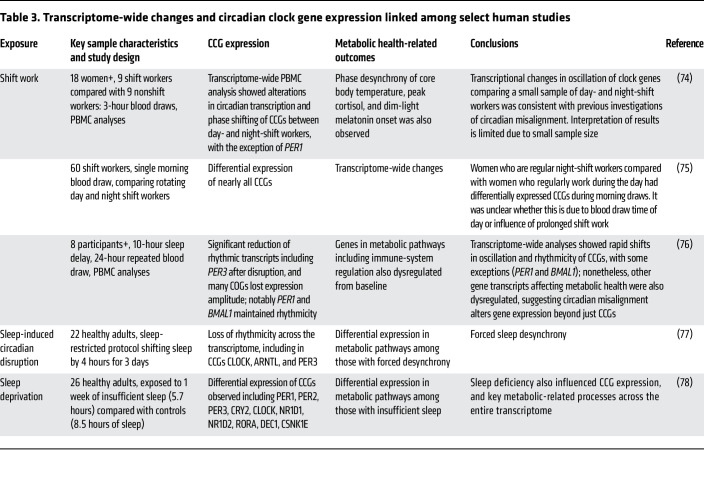
Transcriptome-wide changes and circadian clock gene expression linked among select human studies

**Table 2:**
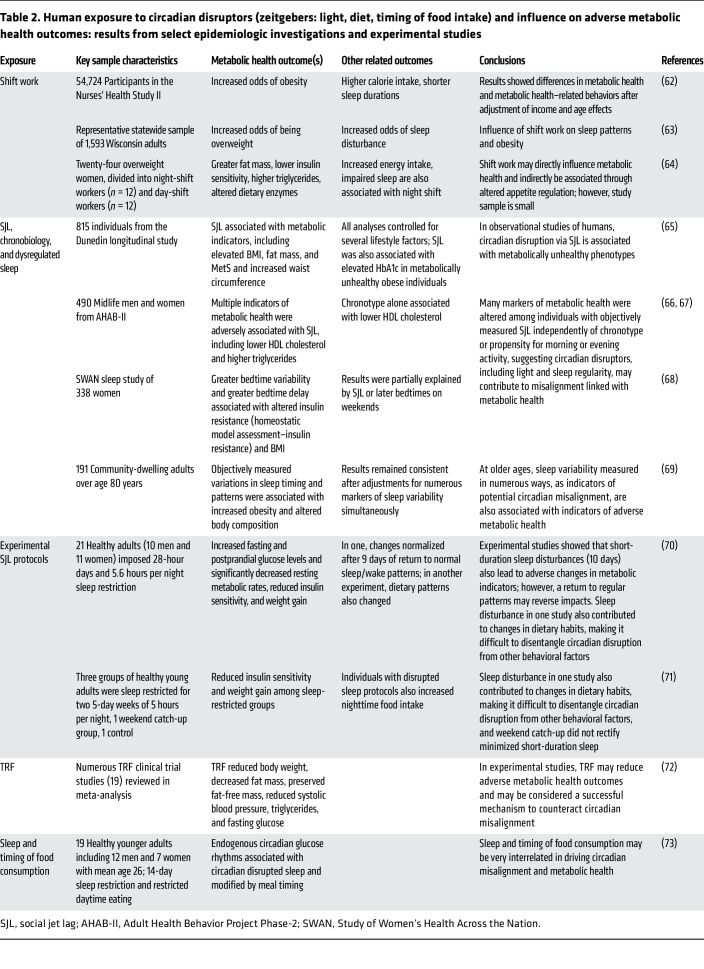
Human exposure to circadian disruptors (zeitgebers: light, diet, timing of food intake) and influence on adverse metabolic health outcomes: results from select epidemiologic investigations and experimental studies

**Table 1:**
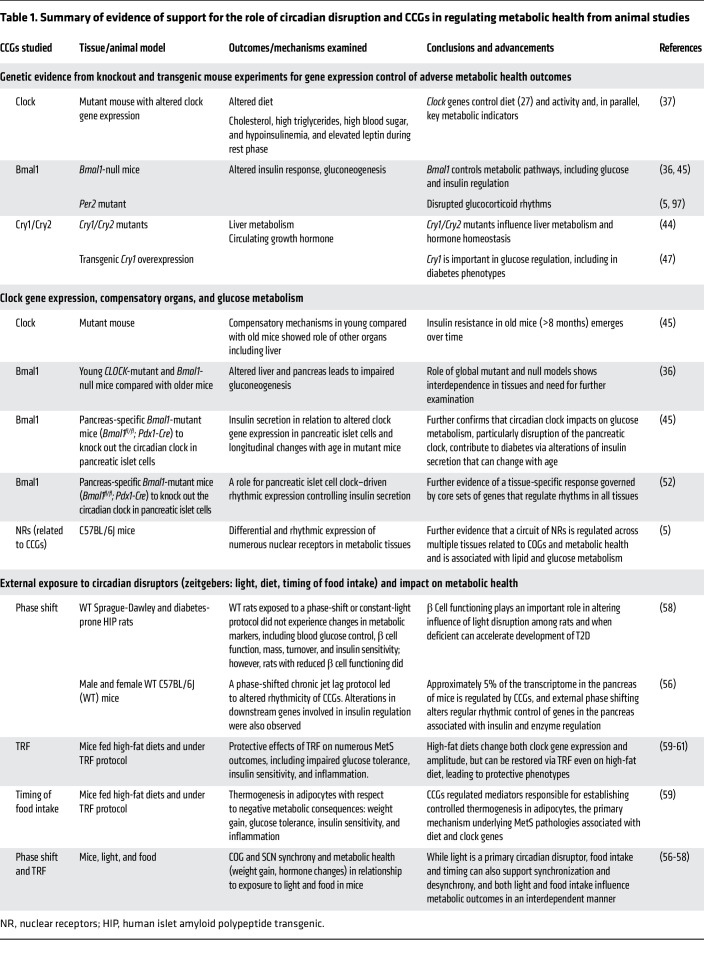
Summary of evidence of support for the role of circadian disruption and CCGs in regulating metabolic health from animal studies

## Version 1 07/15/2024

Electronic publication
